# Number-time interaction: Search for a common magnitude system in a cross-modal setting

**DOI:** 10.3389/fnbeh.2022.891311

**Published:** 2022-08-24

**Authors:** Anuj Shukla, Raju S. Bapi

**Affiliations:** ^1^Cognitive Science Lab, Kohli Centre on Intelligent Systems, International Institute of Information Technology, Hyderabad, Telangana, India; ^2^Thapar School of Liberal Arts and Sciences, Thapar Institute of Engineering and Technology, Patiala, Punjab, India

**Keywords:** ATOM, number-time interactions, time perception and temporal effects, common magnitude system, cross-modal magnitude processing

## Abstract

A theory of magnitude (ATOM) suggests that a generalized magnitude system in the brain processes magnitudes such as space, time, and numbers. Numerous behavioral and neurocognitive studies have provided support to ATOM theory. However, the evidence for common magnitude processing primarily comes from the studies in which numerical and temporal information are presented visually. Our current understanding of such cross-dimensional magnitude interactions is limited to visual modality only. However, it is still unclear whether the ATOM-framework accounts for the integration of cross-modal magnitude information. To examine the cross-modal influence of numerical magnitude on temporal processing of the tone, we conducted three experiments using a *temporal bisection task*. We presented the numerical magnitude information in the visual domain and the temporal information in the auditory either simultaneously with duration judgment task (Experiment-1), before duration judgment task (Experiment-2), and before duration judgment task but with numerical magnitude also being task-relevant (Experiment-3). The results suggest that the numerical information presented in the visual domain affects temporal processing of the tone only when the numerical magnitudes were task-relevant and available while making a temporal judgment (Experiments-1 and 3). However, numerical information did not interfere with temporal information when presented temporally separated from the duration information (Experiments-2). The findings indicate that the influence of visual numbers on temporal processing in cross-modal settings may not arise from the common magnitude system but instead from general cognitive mechanisms like attention and memory.

## Introduction

Previous studies have shown that temporal judgments are biased in the presence of non-temporal magnitudes (Xuan et al., 2007; Srinivasan and Carey, 2010; Cai and Connell, 2015; Schwiedrzik et al., 2016; Yamamoto et al., 2016). More specifically, large numerical magnitudes are judged to last longer compared to small magnitudes. Such cross-dimensional magnitude interaction has motivated a theory of magnitude (ATOM). According to ATOM, magnitudes such as space, time, and quantities are processed through a common metric system in the brain (Walsh, 2003). Due to a common metric system, one magnitude dimension interferes with the processing of the other magnitude even though one magnitude dimension is task-irrelevant. For example, participants overestimated the duration when paired with a large numerical magnitude and underestimated when presented with a small numerical magnitude, although the numerical magnitudes were task-irrelevant (Oliveri et al., 2008; Chang et al., 2011; Hayashi et al., 2013b; Cai and Wang, 2014; Rammsayer and Verner, 2014, 2015). Growing evidence from neuroimaging studies has suggested that such cross-dimension magnitude interactions occur in the frontal and parietal regions of the brain (Hubbard et al., 2005; Bueti and Walsh, 2009; Hayashi et al., 2013a; Skagerlund et al., 2016). Based on this evidence, it has been argued that a common magnitude system processes all kinds of magnitude. On the contrary, a handful of studies have found evidence against the generalized magnitude system and suggested that the magnitudes are processed by domain-specific processing mechanisms (Dormal et al., 2006, 2008; Agrillo et al., 2010; Young and Cordes, 2013; Hamamouche et al., 2018).

The interaction between numerosity and time has also been studied using an adaptation paradigm (Tsouli et al., 2019). It is assumed that if number and time are processed through the common magnitude system, adaptation to numerosity would affect the processing of duration, and adaptation to duration would interfere with numerosity processing. Interestingly, the authors observed a unidirectional effect of adaptation to the duration on numerosity but not the other way around. A recent study investigated the influence of serial dependence of irrelevant magnitude dimension on within and between task-relevant magnitude processing (Togoli et al., 2021). This study introduced the serial dependence using a task-irrelevant inducer (numerosity and duration) and presented it before the reference stimuli. The authors argued that the serial dependencies work within the task-relevant magnitude dimension only but not the cross-magnitude dimension. For example, the numerosity inducer affected numerosity perception but not the duration perception. Similarly, the duration inducer affected duration perception but not numerosity perception. Further, it has also been argued that cross- dimension magnitude interaction can be modulated by visuospatial attention (Vicario et al., 2008; Vicario, 2011; Di Bono et al., 2020; Shukla and Bapi, 2020, 2021) or the memory mechanism (Cai et al., 2018). The previous findings compel us to ask a fundamental question as to whether cross-dimension magnitude interactions result from a generalized magnitude system or arise due to differential cognitive processing mechanisms, for example, due to processes such as attention and memory. In the present study, we would go beyond the basic questions and investigate whether ATOM-framework accounts for multimodal cross-dimensional magnitude interaction, specifically for the magnitudes of number and duration of time.

Previous studies have demonstrated the influence of numerical magnitude on temporal processing and noted that a large numerical magnitude would cause overestimation of time compared to a small numerical magnitude. However, it is essential to note that the number-time interaction has been studied extensively while simultaneously presenting numerical and temporal information, predominantly in the visual domain. Therefore, our current understanding of the generalized magnitude system for processing number and time magnitudes is limited to a particular modality. It is largely unknown how a generalized magnitude system integrates the information presented in a cross-modal manner. The idea here is to test whether or how task-irrelevant/relevant magnitude information presented in one modality affects the processing of task-relevant magnitude of another modality. The central question is whether ATOM-framework accounts for cross-modal information integration for number and time.

To investigate this objective, we conducted three experiments using a *temporal bisection task*. We presented numerical magnitude information in the visual domain and temporal information in the auditory either simultaneously with duration judgment task (experiment-1), prior to duration judgment task (experiment-2), and prior to duration but with the numerical magnitude being task-relevant in a dual-task paradigm (experiment-3). We hypothesized that if, according to ATOM, a central representation exists and integrates magnitude-related information across different modalities, then the presentation of the task-irrelevant magnitude information in one modality would affect the processing of magnitude in another modality in all the three experiments. Also, as posited by ATOM, if a common magnitude system processes time and number, the priming of task-irrelevant magnitude in one modality would influence magnitude processing in the other modality. Essentially, the idea is that if magnitudes are processed by a generalized system, priming with large/small task-irrelevant magnitude in one modality should activate the representation *via* a generalized magnitude system and therefore affect the processing of task-relevant magnitude processing in the other modality.

## Materials and methods

### Participants

Seventy-two participants from International Institute of Information Technology, Hyderabad, India (33 females; age range: 22–27 years) participated in the study. The number of participants (i.e., 66) was estimated using G*POWER 3 (Faul et al., 2007). As per the study design, we used the parameters: alpha level = 0.05, Power = 0.95, and effect size = 0.25. We recruited 72, instead of 66 participants, to avoid any possible drop out due to outliers. Participants were randomly assigned to one of the three experimental groups. Out of the 72 participants, 25 participants took part in experiment-1 and experiment-2. Whereas the experiment-3 had 22 participants. All the experimental procedures and methods were performed in accordance with the relevant guidelines and regulations and the study was approved by the Institute Review Board (IRB), International Institute of Information Technology, Hyderabad, India. Informed consent forms were obtained from all the participants, and remuneration was paid for their participation. None of the participants reported any visual or auditory problems.

### Apparatus

The stimuli were presented and controlled using *OpenSesame* stimulus presentation software (Mathôt et al., 2012) on a 17′′ CRT monitor (1,024 × 768 resolutions) running at 100 Hz frame rate.

### Stimulus

We used two kinds of stimuli to study the cross-modal influence—a visual and an auditory stimulus. The numerical information was always presented in the visual domain as numerals, i.e., “1” and “9” displayed on the monitor. The temporal information in the auditory domain was presented as a sound tone. The numerals were presented in black color against a white background. The tone used was based on a sine wave and was 1,000 ms in duration with a frequency of 440 Hz. The sound tone was presented binaurally through JBL headphones from 100 to 900 ms in steps of 100 ms. The volume of the sound was adjusted for each participant as per their comfort.

### Procedure

#### Experiment-1: Simultaneous

**FIGURE 1:**
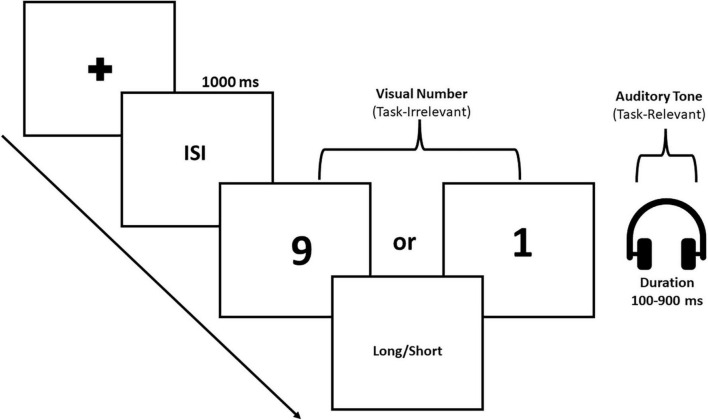
Task-illustration. The trial begins with the fixation cross, followed by the inter stimulus interval (ISI). After the ISI, the numerical information was presented in the visual domain and temporal information in the auditory domain in the form of tone for varied durations. Thus, the numerical and temporal information were presented simultaneously in two different modalities. Participants were required to judge whether the duration of the auditory tone was closer to long or closer to short anchor duration.

Participants were tested in a quiet room. They were asked to sit comfortably. The distance between the participant and the computer monitor was 57 cm. The instruction was given in both verbal and written format. The study took part in three phases—training, feedback, and testing phases. In the *training* phase, the sound tone was presented for 100 and 900 ms as a short and a long anchor duration, respectively. To get a sense of the long and short durations, participants received 10 trials of short and 10 trials of long anchor durations aurally (in the form of tone) along with the numeral “5” on the computer screen. After the training phase, participants were given a *feedback* phase wherein the sound tone was randomly presented either for 100 or 900 ms duration with the numeral “5” displayed on the screen. They were asked to identify whether the tone presented corresponded to the long anchor or to the short anchor duration. Participants were required to respond by pressing the dedicated key for the long/short on the keyboard. Once the response was made, the feedback as *correct* or *incorrect* was presented on the computer screen. In this phase, we ensured that participants performed the duration judgment task with 90% accuracy. Once the participants reached this performance threshold, they were taken to the next phase, i.e., the *testing* phase. In the testing phase, participants were presented a small numerical magnitude or a large numerical magnitude, i.e., “1” or “9” on the visual display and a sound tone was presented in the auditory domain with varying probe durations from 100 to 900 ms durations in steps of 100 ms. Participants were asked to judge whether the presented sound tone was closer to the small anchor or to the long anchor duration they memorized earlier in the training phase. They were asked to press the button “L” on the keypad if they felt the tone duration was closer to the long anchor duration and the button “S” if it was closer to the short anchor duration (see Figure 1). Participants were instructed to judge the tone durations without being influenced by the numerical magnitude presented in the visual domain. Each participants performed a total of 126 trials [2 (Number: 1 and 9) × 9 (Durations: 100 to 900 ms) × 7 (Repetitions)].

#### Experiment-2: Number-time priming

**FIGURE 2:**
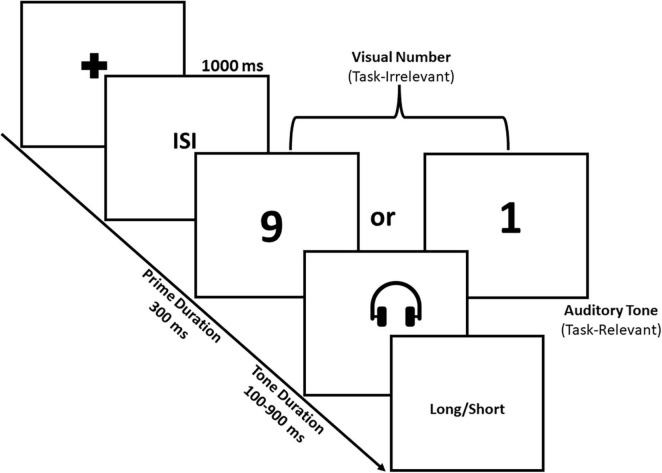
An average psychometric function from each task condition. **(A)** Shows the psychometric plot from the simultaneous condition wherein the numerical magnitudes were presented in the visual domain and duration in the auditory at the same time. **(B)** Shows the psychometric plot from the priming condition wherein the visual numbers were presented for 300 ms in the visual domain prior to the presentation of the duration information in the auditory domain. **(C)** Shows a psychometric fit from the priming dual-task condition where numerical magnitude is used as visual prime and was presented 300 ms prior to the duration task. Unlike the other two tasks, numerical magnitudes were task-relevant in this condition. The red line indicates the fit for the small visual number (“1”), and the green line indicates a fit for the large visual number (“9”).

The stimuli and procedures used in this experiment were like in the experiment-1 except for the testing phase. Unlike in experiment-1, we used a priming paradigm to prime the participants with small and large numerical magnitudes in the visual domain and subsequently presented the sound tone in the auditory domain. Specifically, we presented the numerical magnitudes on the screen for 300 ms followed by the tone for probe durations varying from 100 to 900 ms in steps of 100 ms. Participants were asked to judge whether the presented tone duration was closer to the short anchor or to the long anchor duration memorized during the training phase. In this experiment, we tried to separate the numerical information from that of the temporal. The idea behind such separation is to test whether the numerical and temporal information are processed by a common magnitude system. The assumption behind using the priming paradigm is to activate the common magnitude system while presenting a small and a large numerical magnitude in one modality and study its impact on the temporal processing of the tone in the auditory modality (see Figure 2).

#### Experiment-3: Number-time dual-task priming

The experimental protocols were identical to those in experiment-2 except for a small difference introduced in the testing phase. Unlike in experiment-2, participants were asked to perform the duration judgment task, in a dual-task paradigm, the participants were required to hold the numerical information in the memory while performing the duration judgment task. After the duration judgment, they were required to speak the number presented at the beginning of the trial.

## Results

**FIGURE 3:**
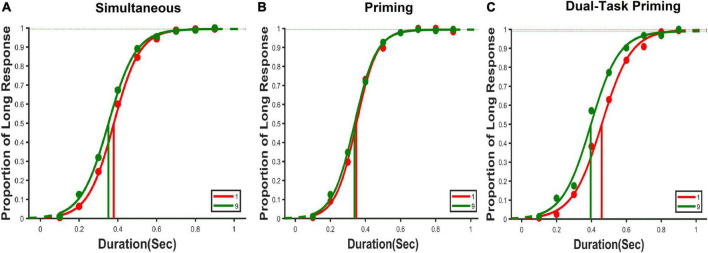
Task-illustration. The trial begins with the fixation cross followed by the 1,000 ms of inter stimulus interval (ISI). After the ISI, the numerical information was presented in the visual domain for 300 ms, followed by the temporal information in the auditory tone. The duration of the auditory tone varied from 100 to 900 ms. Participants were required to judge whether the duration of the auditory tone was closer to long or closer to short anchor duration.

The data were recorded in terms of long and short responses. We used *psignifit-4*, a MATLAB-based toolbox, and estimated a bisection point (BP) for each numerical magnitude condition using a logistic function. The BP is the point at which 50% of the time participants would have perceived the presented duration to be closer to the short anchor and 50% of the time closer to the long anchor duration. The BP is also called as point of subjective equality (PSE) and hereafter we use PSE instead of BP. A left shift of the psychometric curve results in smaller estimates of PSE, whereas a right shift results in larger estimates of PSE. Further, a larger PSE would be interpreted as underestimation of duration and a smaller PSE as overestimation of duration (see Figure 3).

To examine the cross-modal influence of numerical magnitude on temporal processing across different task conditions, we used a 2 (Magnitude: Small and Large) × 3 (Task: Simultaneous, Priming and Priming with Dual-Task) mixed repeated measures ANOVA, wherein Magnitude was a within-subject repeating factor, and task was a between-group factor. Further, the *post hoc* comparisons were made using the *Bonferroni* correction.

The results of the two-way mixed ANOVA revealed that there was a main effect of magnitude [*F*(1,69) = 23.603, *p* < .001, partial η^2^ = 0.255], suggesting that the duration of the tone was overestimated when presented with the large numerical magnitude (360.52 ± 78.39) as compared to when presented with the small numerical magnitude (391.20 ± 82.22). In addition, there was also a significant main effect of task [*F*(2, 69) = 8.223, *p* < 0.001, partial η^2^ = 0.192). The *post-hoc* test indicated that the temporal perception was significantly different for priming with dual-task (Experiment-3) (423.16 ± 74.19) compared to the priming task (Experiment-2) (344.25 ± 72.731) and the simultaneous task (Experiment-1) (365.84 ± 78.36) conditions. However, we did not observe a significant difference in the temporal perception for the priming task (344.25 ± 72.731) compared with simultaneous task (365.84 ± 78.36) conditions.

**FIGURE 4:**
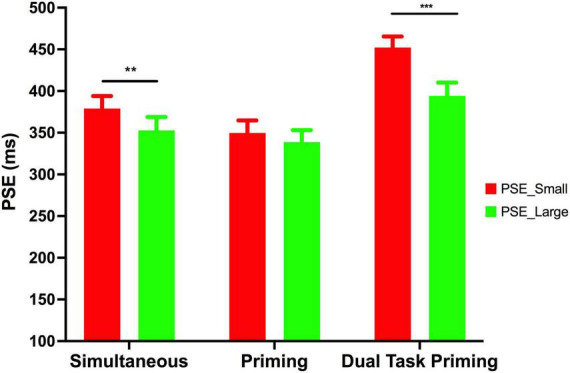
Mean PSE across the task conditions. The red bar shows the mean PSE for the small numerical magnitude (“1”), and the green bar shows the mean PSE for the large numerical magnitude (“9”). The error bar represents the standard error. ** indicates statistical significant differences (*p* < 0.05) *** indicates statistical significant differences (*p* < 0.001).

In addition to the main effect, we also observed a significant interaction between the magnitude and task [*F*(2, 69) = 4.367, *p* < 0.05, partial η^2^ = 0.112] pointing out that the influence of numerical magnitude on temporal processing varied across the task conditions (see Figure 4). Further, the *simple main effect* analysis suggests that the duration of the tone was significantly overestimated for the large numerical magnitude (i.e., “9”) than that for a small numerical magnitude (i.e., “1”) in the priming dual-task condition [*F*(1, 21) = 24.406, *p* < 0.01] and in simultaneous condition [*F*(1, 24) = 4.580, *p* < 0.05]. On the contrary, the temporal perception across different magnitude did not differ in priming condition [*F*(1,24) = 1.252, *p* = 0.247; see Figure 4]. Further, to examine the magnitude of the null result observed in priming experiment, we used *Bayesian paired t-test* using *JASP 0.16.1* to test whether the PSE across the two numerical magnitudes significantly differed from one other. The Bayes factor analysis yields a value of B_10_ = 0.369, considering that it is below one, we can conclude that there is favorable evidence for rejecting the alternative hypothesis (in other words, the results are 2.707 times more likely to have occurred under the null model).

## Discussion

Previous studies have demonstrated that the processing of duration is affected by the presence of numerical magnitudes. Such cross-dimensional interaction has been explained by ATOM advocating for a generalized magnitude system (Xuan et al., 2007; Oliveri et al., 2008; Chang et al., 2011; Hayashi et al., 2013b; Cai and Wang, 2014; Yamamoto et al., 2016). Our study tested the idea of number-time interaction in cross-modal settings and the results suggest that the visual numbers may affect duration judgments of tone only when the number was available at time of temporal judgment. The tone duration was significantly overestimated with a large numerical magnitude compared with the small numerical magnitude presented in the visual domain. The results from experiment-1 and experiment-3 suggest that the numerical magnitude affected the perceived duration of the tone. However, such an effect was not observed in experiment-2 (see Figure 4). Therefore, we suggest that the cross-modal magnitude interaction might occur *via* two possible mechanisms- (a) Interaction *via* working memory: since the numerical and temporal information are presented in two different modalities, these pieces of magnitude information need to be available together for any interaction to take place. Such information integration might take place in the working memory. Therefore, we speculate that cross-modal number-time interaction may occur in the working memory, and attentional mechanism might act as a gatekeeper, preventing task-irrelevant numerical information from getting into the working memory where the temporal processing is already taking place. Thus, the influence of visual number on temporal processing of tone may not be contingent on a common magnitude processing system operating across sensory modalities. A more recent study has already shown that the cross-dimensional magnitude interaction (space-time) arises from memory interference (Cai et al., 2018; Cai and Wang, 2022). (b) Alternatively, explicit processing of numbers may invoke visuospatial processing—it has been shown that the processing of numerical magnitude might elicit a shift of spatial attention which in turn might affect the temporal processing of visual events (Casarotti et al., 2007; Di Bono et al., 2020; Shukla and Bapi, 2021).

In fact, experiment-2 is designed in this spirit where we presented task-irrelevant numerical magnitude in one modality and temporal information in another, assuming that the central representation of a generalized magnitude system could operate based on visually presented numbers either along with duration or with priming cues. We thought that visually presented task-irrelevant numerical magnitude would activate the common magnitude processing system that would influence the subsequent temporal information. Surprisingly, the influence of numerical magnitude on temporal processing disappeared in case of priming (experiment-2) and we did not observe a significant difference in the processing of tone duration across different numerical magnitudes (see Figure 4). Results of experiment-2 indicate that priming with task-irrelevant numerical information did not modulate the representation of duration information. In fact, the finding of experiment-2 seems to be consistent with the findings of Togoli et al. (2021) where the authors have shown a similar effect within the visual modality and suggested that the numerosity of a preceding stimulus does not affect the perceived duration of the current one and vice versa. Such effects have been studied as serial dependence, wherein the sequential effect of a task-irrelevant dimension is studied on the task-relevant dimension. This sequential effect is known to be modulated by factors such as attention (Fischer and Whitney, 2014; Fornaciai and Park, 2018; Fritsche and de Lange, 2019), task relevance (Van der Burg et al., 2019; Togoli et al., 2021), and requires high-level processing (Fornaciai and Park, 2019; Cicchini et al., 2021). In the present study, we used numerals instead of numerosity. The difference between the two is that numerosity is more perceptual in nature, whereas numerals require high-level processing. The present findings may result from the high-level processing and task relevance. It could be possible that the task-irrelevant numerical information might have been filtered out by the attentional system and did not get into the working memory. Thus, visual task-irrelevant numbers did not affect the subsequent temporal processing of tone. Alternatively, it can also be possible that the priming of the numerical information did not activate the common magnitude system. Therefore, no temporal processing difference was observed when primed with small or large numerical magnitudes. The findings from experiment-2 seem to oppose the idea of a central representation of a generalized magnitude for processing time and number when presented cross-modally. Perhaps the generality of such a magnitude processing system is limited to a specific context.

At this juncture, the findings of experiment-3 prove to be interesting and seem to be complementary to the results of experiment-2. When we made the primed number task-relevant in experiment-3, numerical magnitude affected the temporal processing of the tone. This further suggests that the numerical magnitude might be processed and held in working memory along with the temporal information. Therefore, the influence of numerical magnitude on temporal processing of the tone was observed even when the numerical information was temporally separated from the duration judgment task.

Apart from the above, the present result can also be explained by the response bias account (Yates et al., 2012). According to this proposal, the cross-dimensional magnitude interactions arise at the response stage. More specifically, the response tendency for the task-irrelevant magnitude dimension may bias the actual response for the task-relevant magnitude dimension (see also Moon et al., 2015, for a similar account). For example, when judging whether the duration of the auditory tone was long or short, the numerical information was available as “small” or “large” numerical magnitude (experiment-1 and 2). It could be possible that the potential “long” response for the numerical magnitude would prime the “long” response for the duration judgment of the tone. Thus, the numerical magnitude presented in the visual domain might potentially affect the duration judgment of the tone. However, in the case of experiment-2, wherein the numerical magnitude information was task-irrelevant and presented separately from the task-relevant magnitude dimension, it may be possible that the task-irrelevant numerical magnitude did not activate the potential response. Therefore, it did not influence the response of the task-relevant magnitude dimension, in this case, duration judgment of the tone.

The overall findings of the three experimental conditions seem to indicate that task relevance may play an essential role in modulating number-time interactions in cross-modal settings. For example, in experiment-1, the numerical information was task-irrelevant but varied along with the temporal information. Such covariation might have invoked a sense of task relevance; therefore, the numerical information presented in the visual domain affected the temporal processing of the tone. Similarly, in experiment-3, visual numbers were made task-relevant, and participants processed the numbers presented before the duration judgment task. Thus, the task-relevant numerical information of the visual domain modulated the temporal processing of the tone in the auditory domain. On the contrary, in experiment-2, the numerical information presented in the visual domain was task-irrelevant and participants were not explicitly asked to process the number; therefore, the visual numbers did not affect the duration judgment of the tone. Although the findings hinted at the role of task relevance, the present study did not intend to examine the role of task relevance on number-time interaction. Therefore, future studies should investigate the role of task relevance in cross-dimensional magnitude interactions more systematically.

## Conclusion

Our experimental findings suggest that cross-modal numerical and temporal information interacts with each other, and cognitive processes may mediate such cross-dimension magnitude interaction. Both numerical and temporal information should be available in the working memory for cross-modal number-time interaction to take place. Such a system may be limited to visual processing of magnitude information.

## Data availability statement

The raw data supporting the conclusions of this article will be made available by the corresponding author on reasonable request.

## Ethics statement

The studies involving human participants were reviewed and approved by Institute Review Board (IRB), International Institute of Information Technology, Hyderabad, India. The patients/participants provided their written informed consent to participate in this study.

## Author contributions

AS conceptualized and designed the experimental paradigm, collected raw data, as well as analyzed the results, wrote the manuscript, and prepared the figures. AS and RB interpreted the results, revised and reviewed the manuscript, contributed to the article, and approved the submitted version.
